# Functionalizing Diatomite-Based Micro-Arc Coatings for Orthopedic Implants: Influence of TiO_2_ Addition

**DOI:** 10.3390/biomimetics8030280

**Published:** 2023-06-29

**Authors:** Alexander D. Kashin, Mariya B. Sedelnikova, Pavel V. Uvarkin, Anna V. Ugodchikova, Nikita A. Luginin, Yurii P. Sharkeev, Margarita A. Khimich, Olga V. Bakina

**Affiliations:** 1 Laboratory of Physics of Nanostructured Biocomposites, Institute of Strength Physics and Materials Science of SB RAS, Tomsk 634055, Russia; kash@ispms.ru (A.D.K.); uvarkin@ispms.tsc.ru (P.V.U.); ugodch99@gmail.com (A.V.U.); nikishek90@gmail.com (N.A.L.); sharkeev@ispms.ru (Y.P.S.); 2 Laboratory of Plasma Synthesis of Materials, Troitsk Institute for Innovation & Fusion Research, Moscow Region, Troitsk 108840, Russia; 3 Research School of High-Energy Physics, National Research Tomsk Polytechnic University, Tomsk 634050, Russia; 4 Laboratory of Nanobioengineering, Institute of Strength Physics and Materials Science of SB RAS, Tomsk 634055, Russia; khimich@ispms.tsc.ru (M.A.K.); ovbakina@ispms.tsc.ru (O.V.B.)

**Keywords:** orthopedic implants, magnesium alloy, micro-arc coating, diatomite, TiO_2_, in vitro biocompatibility, corrosion resistance, adhesive strength

## Abstract

The method of micro-arc oxidation has been utilized to synthesize a protective biocompatible coating for a bioresorbable orthopedic Mg implant. This paper presents the results of comprehensive research of micro-arc coatings based on diatomite—a biogenic material consisting of shells of diatom microalgae. The main focus of this study was the functionalization of diatomite-based micro-arc coatings by incorporating particles of titania (TiO_2_) into them. Various properties of the resulting coatings were examined and evaluated. XRD analysis revealed the formation of a new magnesium orthosilicate phase—forsterite (Mg_2_SiO_4_). It was established that the corrosion current density of the coatings decreased by 1–2 orders of magnitude after the inclusion of TiO_2_ particles, depending on the coating process voltage. The adhesion strength of the coatings increased following the particle incorporation. The processes of dissolution of both coated and uncoated samples in a sodium chloride solution were studied. The in vitro cell viability was assessed, which showed that the coatings significantly reduced the cytotoxicity of Mg samples.

## 1. Introduction

In recent years, a lot of scientific research has aimed to develop new materials and designs for the creation and improvement of bone implants [1,2]. Much attention is paid to ensuring mechanical strength, fatigue durability of the implant, and reducing its weight [3,4]. Magnesium and its alloys have become increasingly popular in the field of medical materials science. In particular, magnesium can be used as a material for the fabrication of bioresorbable orthopedic implants. This is feasible due to its low specific gravity (≈1.74 g/cm^3^), high biocompatibility, and elastic modulus close to that of human cortical bone (≈40–45 GPa) [4,5,6,7,8,9,10]. Such a modulus of elasticity virtually eliminates the risk of the stress-shielding effect, which can lead to undesirable consequences in the form of the onset of osteopenia at the implant site [11,12,13]. According to Wolff’s law, the bone in a healthy human or animal adapts to the stresses to which they are subjected [14]. If the load on the bone decreases, the bone becomes less dense and weaker due to the lack of incentive needed to continue remodeling. Therefore, magnesium is a very promising material in this respect.

However, magnesium also has certain disadvantages associated with its excessive susceptibility to corrosive media, as well as the active release of hydrogen at the site of contact between the implant and the surrounding tissues [15,16,17,18]. To address these challenges, this paper proposes modifying the implant surface with a protective, biologically active coating based on natural diatomite functionalized with titanium dioxide particles.

To synthesize the coatings, the micro-arc oxidation (MAO, also known as plasma electrolytic oxidation or PEO) method was implemented. This method is characterized by coating a valve metal substrate submerged in a bath of electrolytes. Under the influence of a strong electric field, numerous plasma micro-arc discharges are formed on the surface of the substrate, contributing to intensive electrochemical interaction of the electrolyte components with the substrate material, and consequently, the formation of a protective oxide coating [19,20]. This coating acts as a two-way solution. On the one hand, it protects the magnesium implant from being dissolved too quickly by creating a barrier between it and the aggressive environment [21,22,23,24]. On the other hand, it enables and facilitates the osseointegration process between the implant and the bone tissue [25,26,27,28,29].

The most widely used coatings in the field of micro-arc coating research are the ones synthesized in electrolytes based on phosphates, silicates, and, aluminates, although these are a little less common [30,31,32,33,34,35,36,37]. Aluminate-based electrolytes are typically used to produce wear-resistant MAO coatings due to the dominant formation of MgAl_2_O_4_ as a very solid phase. Phosphate- and silicate-based electrolytes are commonly used to synthesize coatings with improved corrosion resistance, which is due to the high corrosion resistance of Mg_2_SiO_4_ and Mg_3_(PO_4_)_2_, respectively. [38]. Such coatings can be further functionalized by the addition of insoluble particles to the composition of an electrolyte. These can include various pharmaceutical products, carbides, nitrides, oxides, etc. [39,40,41,42,43,44,45,46,47]. Altering the concentration and/or proportions of these particles makes it possible to control and tailor the structure and various properties of the resulting coatings.

Another promising direction is the use of materials of biogenic origin to increase the osteoinductive/osteoconductive properties of bone-contact implantable devices, which contributes to their further osseointegration. In previous studies, we developed coatings for a magnesium alloy based on natural diatomite [48], a sedimentary rock of biogenic origin consisting of skeletons of diatom algae. The mineralogical composition of diatomite is mainly represented by amorphous hydrated silica SiO_2_∙nH_2_O. Diatomite powder was introduced into the composition of the electrolyte to form micro-arc coatings. The obtained coatings were characterized by excellent biocompatibility and high corrosion resistance. However, the mechanical properties of these coatings were not outstanding.

In this work, an electrolyte based on biogenic diatomite was used as the base electrolyte. The distinction of this work is the modification of a diatomite-based coating with titanium oxide particles added to the base electrolyte in the form of a dispersed phase. Analysis of the scientific literature on this topic shows that the addition of titanium dioxide particles changes the structure and properties of micro-arc coatings, increasing their adhesive strength, corrosion resistance, wear resistance, and antibacterial properties [49,50,51,52,53,54]. Ergo, the addition of TiO_2_ particles is a promising direction in the field of the functionalization of micro-arc coatings. The novelty of this work lies in the fact that completely new composite coatings based on biogenic diatomite with the addition of TiO_2_ particles characterized by high biocompatibility, adhesive strength, and excellent corrosion resistance were developed.

The main objective of this study is to investigate the effect of adding refractory titanium oxide particles on the structure and various properties of protective, biologically active micro-arc coatings for bioresorbable orthopedic magnesium implants.

## 2. Materials and Methods

As a substrate material, high-purity magnesium alloy (Mg alloy) MA2-1hp (AZ31 alloy equivalent) (VILS, Moscow, Russia) was chosen. Its chemical composition is as follows (wt.%):

- 94.12 Mg;
- 4.05 Al;
- 1.1 Zn;
- 0.6 Mn
- >0.1 other elements combined.

The alloy has a Young’s modulus of 42 GPa and a density of 1.790 kg/m^3^, based on the manufacturer’s tests. Model samples for the coating deposition were cut from a solid piece of magnesium alloy by the method of electric discharge machining. After that, the samples were ground with 600-grit abrasive sandpaper (Hermes Schleifmittel GmbH, Germany) to achieve surface roughness Ra of 0.5–0.6 µm. Following this, the samples were deep-cleaned using an ultrasonic cleaner (Elmasonic S, Elma, Germany) filled with distilled water and ethanol sequentially for 30 min each. After this preparation, samples with a smooth surface were obtained. When examining uncoated samples with an optical or electron microscope, distinctive scratches were observed on their surface, which remained after the mechanical impact of the abrasive material.

**Figure 1:**
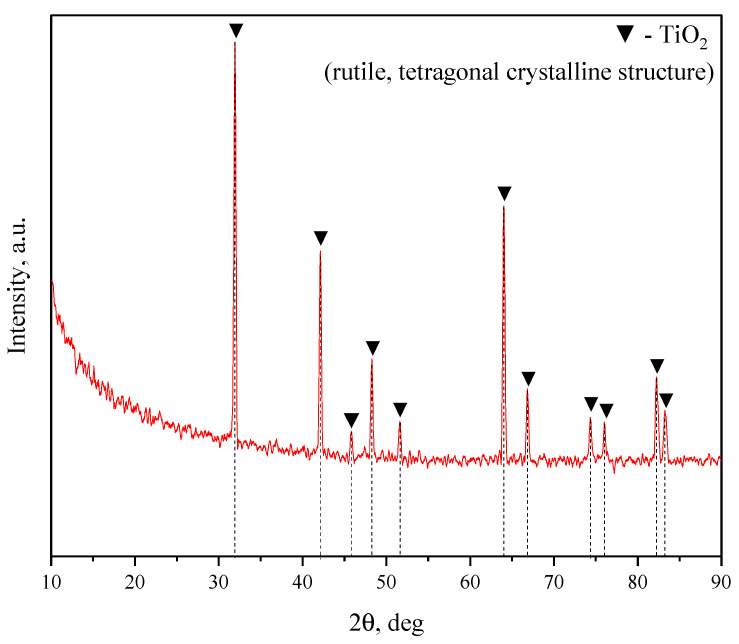
X–ray diffraction pattern of the initial TiO_2_ powder.

The electrolyte for the coating deposition included NaOH, Na_2_SiO_3_, and NaF dissolved in distilled water and dispersed phases of diatomite (SiO_2_∙nH_2_O) and titania (TiO_2_). The diffractogram of TiO_2_ powder is shown in Figure 1. The diffractogram of diatomite powder was provided previously in [48].

The coatings were synthesized via the method of micro-arc oxidation. For the coating deposition process, the anodic potentiostatic mode was implemented with a pulse frequency and duration of 50 Hz and 100 µs, respectively. The coatings were deposited for 5 min at four different pulsed voltages: 350, 400, 450, and 500 V. To carry out the experiment, “Micro-Arc 3.0” equipment was used, which consisted of a direct current (DC) power supply, a water-cooled electrolyte bath, and counter electrodes. The monitoring of the parameters during the MAO process was carried out with the help of the equipment’s built-in software. A magnetic mixer stirred the electrolyte continuously during the process to agitate the solution and prevent particle aggregation. After MAO treatment, samples with a uniformly distributed coating layer were obtained.

The surface morphology and the elemental composition of the resulting coatings were studied using a LEO EVO 50 scanning electron microscope (Zeiss, Jena, Germany), equipped with an INCA x-act EDX spectrometer (Oxford Instruments, Abingdon, UK).

Changes in the phase composition of the coatings depending on the deposition voltage were investigated via an X-ray diffractometer (DRON–7, Burevestnik, St. Petersburg, Russia) with the following parameters: CoK_α_ radiation; 35 kV voltage; 22 mA tube current; and 2*θ* angle range of 10° to 90° with 0.02° scanning step.

The adhesion strength of the coatings was assessed using a Revetest RST scratch tester (CSM Instruments SA, Peseux, Switzerland) equipped with a diamond indenter 200 µm in radius. The indenter scratched the surface of the sample, moving at a speed of 1 mm/min, while the load increased linearly. The length of the scratch was 5 mm. The peak indentation force was 40 N, and the loading rate was 3.9 N/min.

Corrosion resistance of the coatings in comparison with uncoated Mg alloy was determined using the “P-40X” electrochemical workstation (Electrochemical Instruments, Russia). A 0.9% NaCl solution was used as the electrolyte in a three-electrode cell, where a graphite rod served as a counter electrode and an Ag/AgCl electrode was used as a reference electrode. The exposed sample surface area equaled 1 cm^2^. The scan rate at which the potentiodynamic polarization curves were obtained equaled 2 mV/s. The electrode potential ranged from −1.9 V to +1.9 V. The temperature of the electrolyte was maintained at 37 °C to simulate the conditions under which a coated implant would function in a human body.

The method of immersion was used to determine the biodegradation rate of both the coated and uncoated Mg alloy specimens. The samples were placed in containers with 0.9% NaCl physiological saline solution and immersed at 37 °C in compliance with ISO 10993-5. The volume of the electrolyte was at least 1 mL per 1 cm^2^ of the sample surface and covered the sample completely. To obtain reliable results, the electrolyte was prepared under aseptic conditions. The experiment was conducted over the course of 11 days. The number of parallel experiments was not less than 3. Mass loss of the samples during dissolution was monitored on a daily basis. The following formula (1) was used to calculate the mass loss of the specimens:

(1) $$∆m=\frac{m_{0}-m_{i}}{m_{0}}\cdot 100\%$$

where *m*_0_ is the sample mass before dissolution, mg, and *m_i_* is the sample mass after dissolution, mg.

The condition of the coatings at certain stages of the experiment was visually assessed via a MET 1MT optical microscope (Altami, Russia).

The biocompatibility of samples was examined in vitro using the MTT assay after direct contact of the cells with the testing material. The NIH/3T3 cell line was purchased from the State Research Center of Virology and Biotechnology “VECTOR” (Novosibirsk, Russia). The cells were seeded in a flask with DMEM (Lonza, Switzerland) with 10% fetal bovine serum (HyClone, Marlborough, MA, USA) and 5% penicillin/streptomycin-glutamine (HyClone, Marlborough, MA, USA) and incubated at 37 °C in a 5% CO_2_. After the 24 h incubation period, the attached cells were trypsinizated for 3–5 min and centrifuged at 1400 rpm for 5 min. The cell culture was seeded into 24-well culture plates (total volume of 2 mL) at 70,000 cells per well. Test samples were placed in each well. Cells were incubated for 24 h at 37 °C in 5% CO_2_. Cells that were incubated in the medium without the sample present were used as controls.

The viability of the cells after exposure was analyzed using the MTT assay [48]. In line with typical procedure, 200 µL of MTT [3-(4, 5-dimetheylthiazol-2)-2, 5 diphenyl tetrazolium bromide] solution was added to each well. The plates were incubated for 4 h at 37 °C in 5% CO_2_. Then, the MTT-containing medium was removed, and the cells were treated by adding 100 μL DMSO (HyClone, Marlborough, MA, USA) to dissolve the formazan crystal. In the final step, the optical density (OD) of the solution was recorded at a 570 nm wavelength using a microplate reader Thermo Scientific Multiskan FC (Thermo Fisher Scientific, Waltham, MA, USA). The measured OD value was used to calculate the relative cell viability (%) according to Equation (2):

Cell viability (%) = OD (test sample)/OD (control) × 100(2)

Each experiment was carried out in duplicate. All statistical analyses were performed using the statistical software package STATISTICA 10.0. The normal distribution of the results was checked by the Kolmogorov–Smirnov test. The significance of differences in the mean values between groups was analyzed using the Mann–Whitney test. Differences were considered significant at *p* < 0.05.

## 3. Results and Discussion

### 3.1. Coating Thickness and Roughness

**Figure 2:**
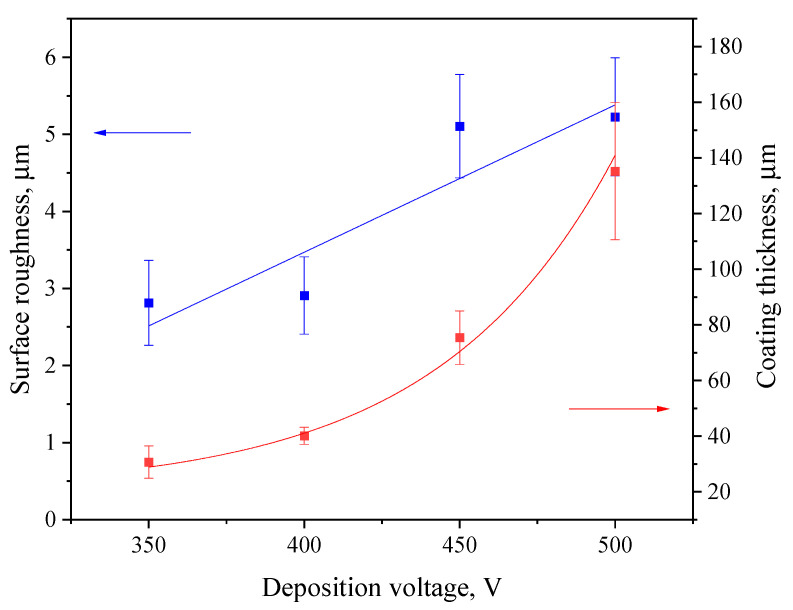
Graphs of roughness (left axis, blue line) and thickness (right axis, red line) versus the MAO process voltage.

Figure 2 displays the thickness and roughness of the coatings in relation to the MAO process voltage. The thickness of deposited coatings increases exponentially, which correlates well with our previous studies related to diatomite-based coatings [48,55]. The surface roughness ranges from 2.5 to 5.0 µm, with a sharp increase at 450 V. This is due to the more intense course of the micro-arc oxidation process at higher voltages, which leads to a greater number of micro-arc plasma discharges on the substrate surface. This, in turn, leads to the merging of many small micro-discharges into one large electric arc, leading to more active intumescence of the surface and, accordingly, to the formation of a rougher topography.

### 3.2. Morphology and Pore Structure

**Figure 3:**
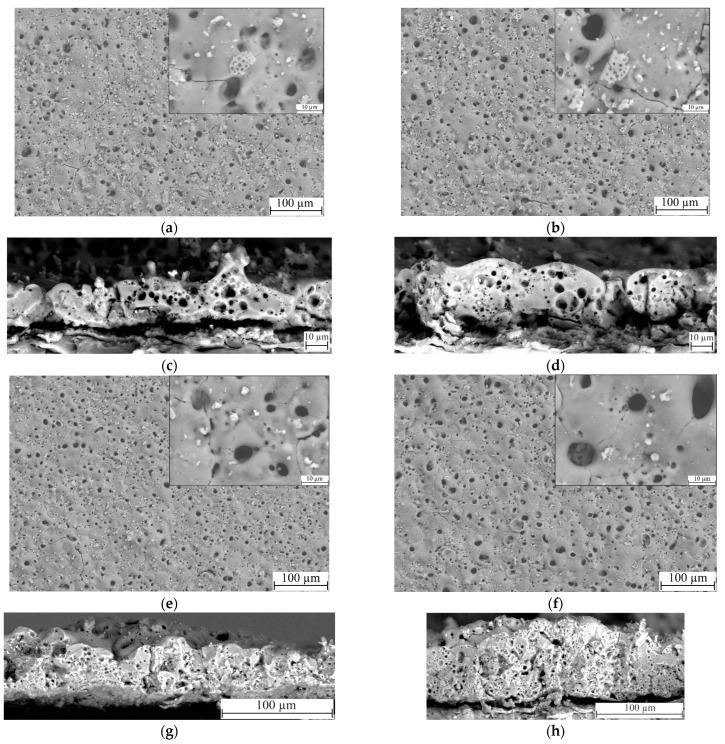
SEM micrographs of the surfaces and cross-sections of TiO_2_-doped diatomite coatings synthesized at different process voltages: (**a**,**c**) 350 V; (**b**,**d**) 400 V; (**e**,**g**) 450 V; (**f**,**h**) 500 V. Mag.: 500×, 5000× for (**a**,**b**,**e**,**f**); 2000× for (**c**,**d**); 1000x for (**g**,**h**).

Visual analysis of the SEM micrographs of the coatings obtained at different process voltages was carried out (Figure 3). It was revealed that the coatings have a large number of variously shaped pores, ranging in size from 100 nm to 12 µm (Figure 3a,b,e,f). We can observe the fragments of lithified diatomeae shells both on the surface and within the bigger pores. Some of the fragments were partially melted into the matrix of the coating, thus giving it an ordered reticulated pore structure. Unmelted particles of both diatomite and titania are also present in substantial amounts on the surface of the coating. It is worth noting, however, that the number of unmelted particles decreases with the increase in the deposition process voltage. This can be explained by a larger quantity of more powerful micro-arc discharges being generated during the MAO process at higher voltages, which leads to a more active disintegration and fusion of the oxide particles into the coating matrix.

In addition, Figure 3c,d,g,h presents the micrographs of cross-sections of the coatings. The analysis of the microphotographs leads to the conclusion that the coatings have an internal porous structure, including pores of different types and sizes. The coatings deposited at MAO voltages of 350 and 400 V contain large pores, up to 5 μm in diameter, indicating single high-intensity micro-arc discharges (Figure 3c,d). Groups of small pores less than 1 μm in size are scattered around them, resulting from cascades of micro-arc discharges. As the voltage of the MAO process increases to 450–500 V, the thickness of the coatings increases significantly, but the nature of the internal pore structure does not change; both large and small pores, evenly distributed throughout the coating thickness, are present in the coatings (Figure 3g,h). The pores are mostly enclosed, but in some cases channeled pores resulting from a powerful breakdown of the coating are observed (Figure 3g). In addition, the presence of TiO_2_ particles can be observed in the cross-sections of the coatings.

### 3.3. Elemental Composition

**Figure 4:**
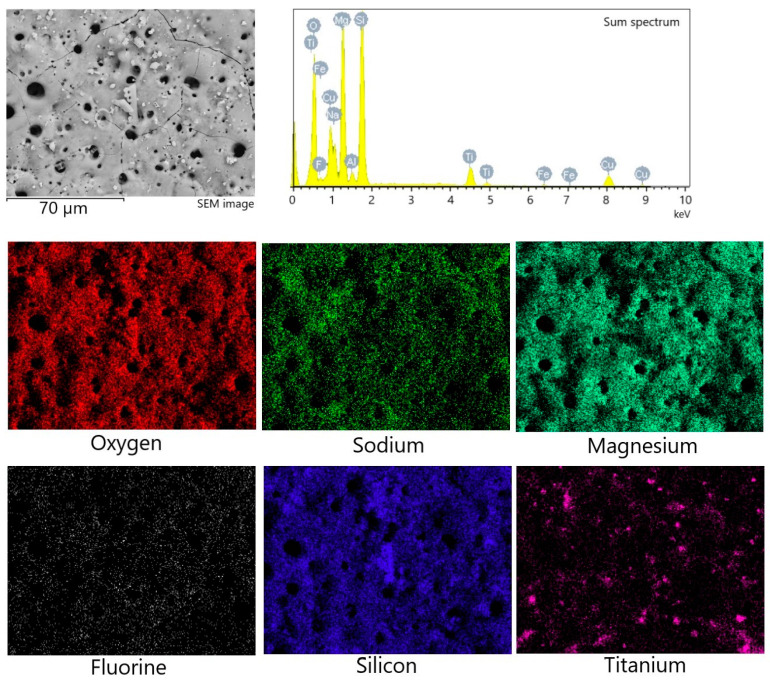
Energy-dispersive X-ray analysis of the TiO_2_-incorporated diatomite coating formed at 500 V: SEM image, sum spectrum, and element mapping.

To characterize the chemical composition of the synthesized coatings, the method of energy-dispersive X-ray microanalysis was used. Figure 4 shows the maps of element distribution, as well as the sum spectrum of the elements present in the coating. High localized concentrations of Ti and Si were detected via element mapping, correlating to titania and diatomite particles, respectively. Table 1 displays the amount of each element (in at. %) in relation to the coating deposition voltage. It can be concluded that the amount of most elements remains approximately the same regardless of the process voltage. The exceptions are silicon and titanium, the amount of which decreases as the MAO voltage increases. This can be partially attributed to fewer particles of both diatomite and titania remaining unmelted on the coatings surface at higher MAO voltages, as can be seen in Figure 3.

**Figure 5:**
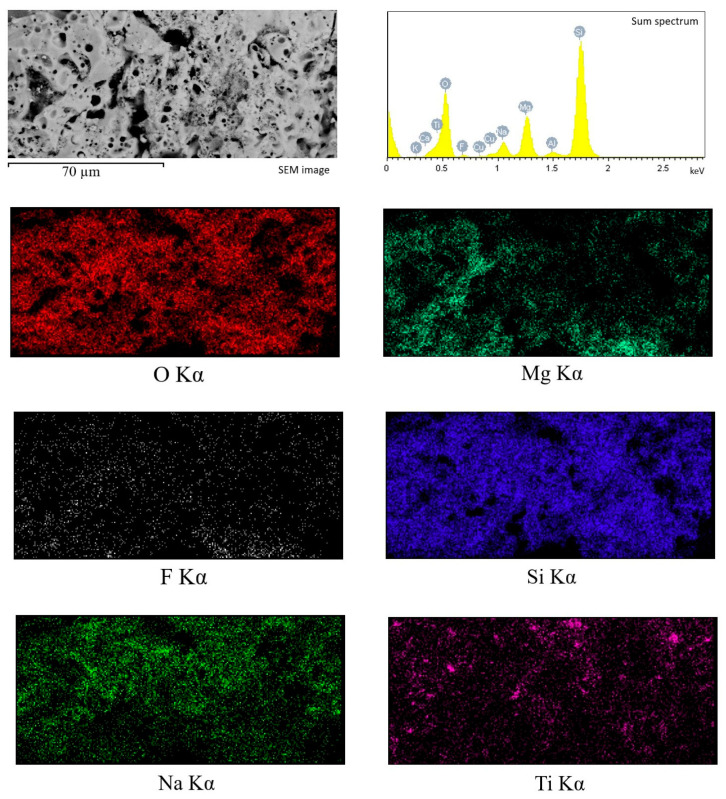
Energy-dispersive X-ray analysis of the cross-sections of TiO_2_-incorporated diatomite coating formed at 450 V: SEM image, sum spectrum, and element mapping.

EDX analysis of the element distribution in the cross-section of the coating (Figure 5) showed that the elements O and Si are evenly distributed throughout the coating thickness, while Mg and F are mainly concentrated closer to the substrate in the transition layer. Na concentration, on the contrary, is higher in the surface layer. In addition, isolated local Ti accumulations are observed, confirming the presence of TiO_2_ particles in the coating volume.

The chemical composition of the cross-sections of the coatings formed at different voltages of the MAO process (Table 2) is almost identical to the composition of the surface of these coatings presented in Table 1. In the cross-sections of the coatings applied at voltages of 350–400 V, a higher content of F is noted, but with an increase in voltage, its amount decreases. There is also a decrease in Mg concentration when the process voltage increases to 450–500 V.

#### X-ray Diffraction Analysis

**Figure 6:**
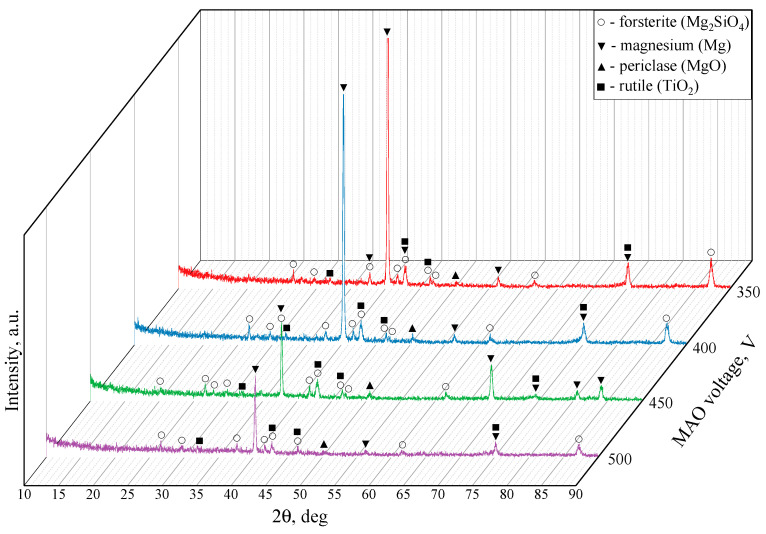
X-ray diffraction patterns of the coatings formed at 350, 400, 450, 500 V.

Figure 6 showcases the X-ray diffraction patterns for the TiO_2_-augmented diatomite coatings formed at four different process voltages. The formation of a new predominant phase is detected in all four coatings, namely forsterite (Mg_2_SiO_4_) (ICDD #34-0189), which is a magnesium orthosilicate from the olivine group of minerals. We can also see the reflexes correlating to both magnesium (ICDD #35-0821) and its oxide, namely periclase (ICDD #45-0946). The incorporation of TiO_2_ particles into the coating results in a few peaks corresponding to rutile (ICDD #21-1276), which is the most common natural form of titanium dioxide.

### 3.4. Mechanical Properties

#### Scratch Testing

**Figure 7:**
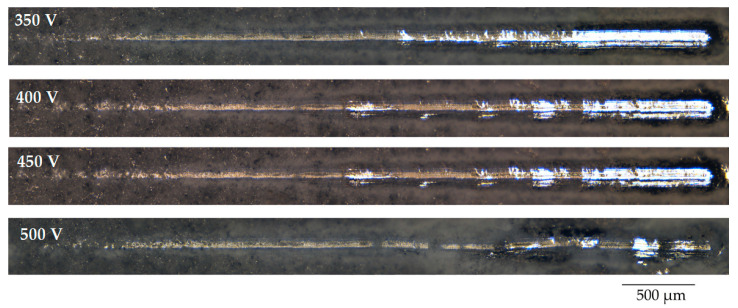
Optical images of the scratches obtained as a result of testing the coatings deposited at different MAO voltages.

The results of the study of the mechanical properties of TiO_2_-augmented diatomite coatings, performed by scratch testing, showed that they have very high adhesion strength. Figure 7 shows the optical images of the coatings, applied at different voltages of the MAO process, after scratch testing. On scratches, it is possible to distinguish the zone of destruction of the coating at the moment of penetration of the indenter to the substrate—this is a solid light zone at the end of the scratch. But for the coatings applied at 500 V, this zone is practically absent, which indicates the highest adhesion strength of these coatings.

**Figure 8:**
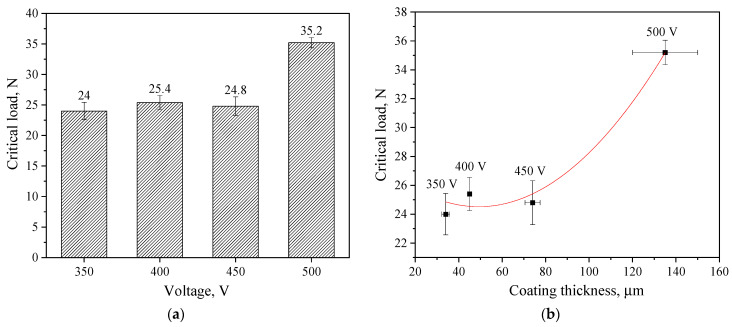
Diagram of correlation between the critical load and the MAO voltage (**a**); graph of the correlation dependence of the critical load on the thickness of coatings formed at different voltages (**b**).

The determination of the critical load at failure confirmed these results. A diagram of the critical load versus voltage at which the coating was formed is shown in Figure 8a. The figure shows that the critical load values for coatings applied at 350–450 V are approximately the same, ranging from 24.0–25.4 N. However, the coating applied at 500 V is characterized by the highest critical load value of 35.2 N. The results obtained in previous studies show that diatomite-based coatings without TiO_2_ particles have much lower adhesion strength. The maximum critical load did not exceed 10 N [48].

Because the thickness of the coatings increases exponentially with an increase in the stress of the MAO process, we assume that the adhesive strength of the coatings depends on their thickness.

Figure 8b shows a correlation graph of the dependence of the critical load on the thickness of the coatings, indicating that this dependence has an exponential character, which confirms our assumption.

### 3.5. Electrochemical Properties

**Figure 9:**
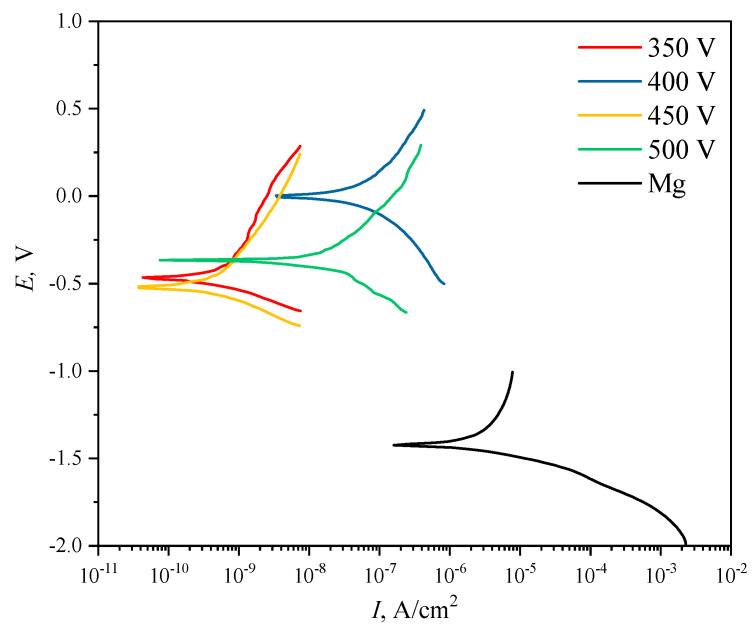
Potentiodynamic polarization (PDP) curves of the studied samples.

One of the main objectives of this study was to improve the corrosion properties of diatomite-based coatings by incorporating titanium oxide particles into them. This is especially relevant for aggressive media, particularly the liquid component of the physiological medium of the human body. Figure 9 shows the potentiodynamic polarization curves for both pure Mg alloy samples and samples with coatings applied at different voltages. The values of polarization resistance and corrosion current density of the coatings varied in the ranges from 1.65 × 10^6^ to 1.41 × 10^8^ Ω cm^2^ and from 4.01 × 10^−8^ to 3.30 × 10^−10^ A cm^–2^, respectively, versus MAO process voltages (Table 3). Comparison with the literature data [56,57] shows that calcium-phosphate coatings obtained by the PEO method on magnesium alloys are characterized by lower values of polarization resistance in the range of 4.24–11.27 kΩ cm^−2^, and higher values of corrosion current density are characterized by the range of 1.25 × 10^−6^–8.54 × 10^−6^ A cm^−2^. This indicates that these coatings are inferior in corrosion resistance to diatomite-based coatings with TiO_2_ particles as presented in this paper.

It can be seen that the coatings deposited at 350 and 450 V are the most resistant to corrosion. The values of polarization resistance R_p_ for these samples amounted to 1.22 × 10^8^ A∙cm^−2^ and 1.41 × 10^8^ A∙cm^−2^, respectively.

A comparative analysis of the corrosion resistance of diatomite coatings before [48] and after the addition of titanium dioxide particles showed that the addition of particles increases the polarization resistance of coatings by 2–3 orders of magnitude, depending on the coating deposition voltage. The corrosion current density in this case decreases by 1 to 2 orders of magnitude. Overall, the corrosion current density of TiO_2_-doped samples decreases by 2–4 orders of magnitude compared to uncoated Mg alloy samples, and the polarization resistance, respectively, increases by 2–4 orders of magnitude. The experimental data are given in Table 3.

### 3.6. Bioresorption Study

**Figure 10:**
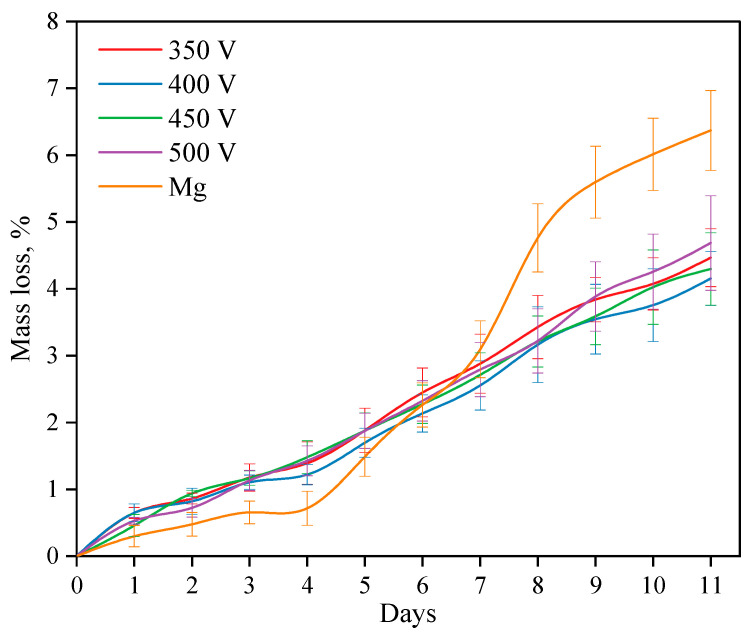
Graph of the samples’ mass loss depending on the time of immersion in the 0.9% NaCl solution.

The processes of dissolution of both coated and uncoated alloy samples were studied for 11 days while kept in a 0.9% NaCl solution (Figure 10). Studies have shown that when a Mg alloy is dissolved, three periods can be distinguished. During the first period, i.e., during the first 4 days, the dissolution rate of the alloy is lower than the dissolution rate of samples with coatings. After 4 days, the dissolution rate increases sharply and exceeds the dissolution rate of the coated samples. After 8 days, the dissolution rate slightly decreases again. As described in previous studies, the dissolution rate of the Mg alloy directly depends on two processes: the first is the dissolution of alloy components, while the second is the process of reverse precipitation of dissolution products (magnesium hydroxide Mg(OH)_2_) on the alloy surface. If the first process prevails, the dissolution rate increases, but if the second process prevails, the alloy dissolves more slowly.

The rate of dissolution of coatings of all types is constant and linear. After 6 days of dissolution, the mass loss of the coated samples becomes less than that of the Mg alloy. The intensity of the dissolution of the coating does not depend on the voltage of the MAO process at which the coating is applied.

**Figure 11:**
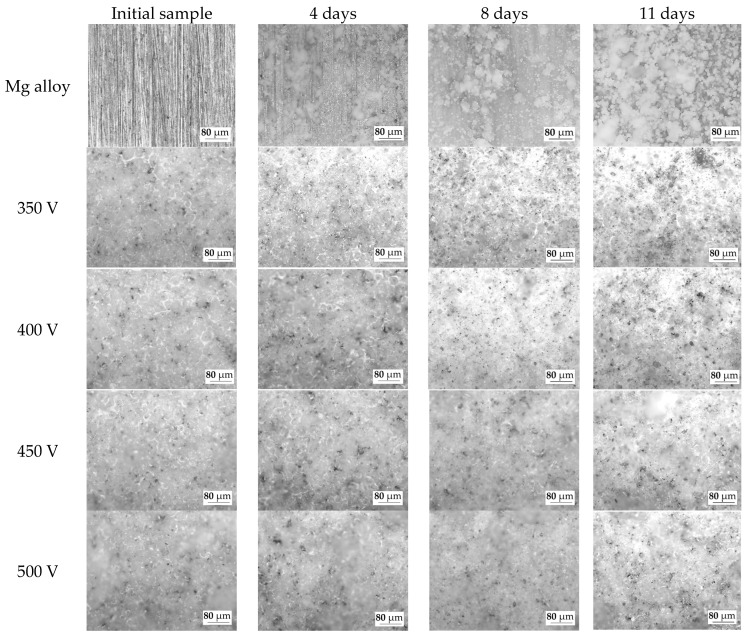
Optical microscopic images of the samples at different stages of the bioresorption process.

The optical images of the samples before dissolution and after 4, 8, and 11 days of dissolution were also analyzed (Figure 11). On the surface of the pure Mg alloy, the precipitation of dissolution products is observed after 4 days of incubation in the 0.9% NaCl solution. The amount of dissolution products on the surface of the Mg alloy increases with the increase in the duration of incubation. Similar patterns were observed and described in previous studies [48,55].

As the optical images show, the dissolution of the coatings occurs in the area of surface structure defects (pores, cracks). An increase in the diameter and depth of the pores can be noted. But the morphology of the coatings surface does not change significantly, which indicates the resistance of this type of coatings to dissolution in the biological environment. This phenomenon is explained by the presence of insoluble compounds, such as magnesium silicate (forsterite) and oxides of magnesium and titanium, in the coatings.

### 3.7. In Vitro Cytotoxicity

**Figure 12:**
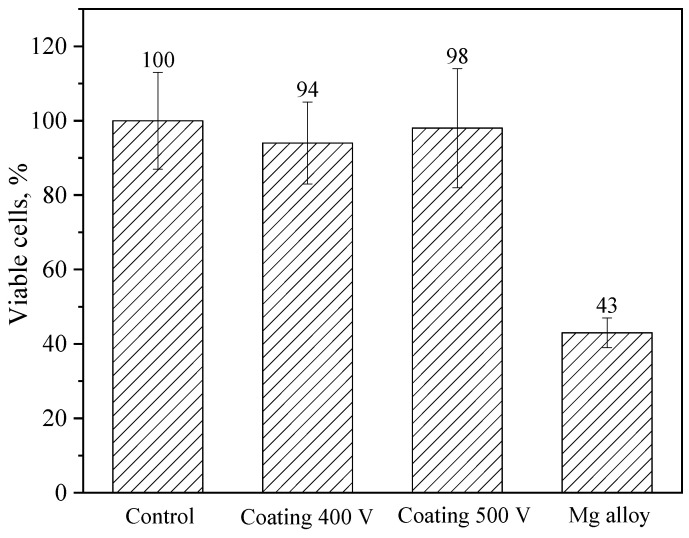
Results of in vitro NIH/3T3 cell viability measured by an MTT assay after 24 h culturing with negative control (DMEM), as well as with Mg alloy (positive toxic control), or samples coated at 400 V and 500 V.

Cell viability was assessed using the MTT assay and quantified according to Equation (2). Compared to the control, coating 400 V and coating 500 V-treated cells showed no statistically significant decrease in viability; cell viabilities were about 94% and 98% of that of the control, respectively (Figure 12). At the same time, the Mg alloy-treated NIH/3T3 cells showed decreased viability, and the cell viability was approximately 43% (high toxicity). Based on these results, it is safe to say that the coatings significantly reduce the toxicity of the samples.

## 4. Conclusions

The coatings considered in this article are suitable for use as a biocompatible and bioactive protectant for magnesium-based bioresorbable orthopedic implants. The micro-arc oxidation method was used to synthesize coatings based on diatomite with the addition of titanium dioxide particles. A range of 350 to 500 V was chosen as the optimal voltage range of the MAO process. Depending on the MAO voltage, the thickness of the coating varied from approximately 30 to 140 µm, and the roughness values lay within a 2.5–5.0 µm range.

The resulting coatings were studied using a number of research methods, including scanning electron microscopy (SEM), energy-dispersive X-ray microanalysis (EDX), X-ray diffraction analysis (XRD), scratch testing, potentiodynamic polarization testing, in vitro immersion method, and in vitro MTT assay.

It has been found that the morphology of the coatings is comprised of a large number of pores of various shapes and sizes, fragments of lithified diatomeae shells, and unmelted particles of both diatomite and TiO_2_. Some diatom algae fragments were partially fused into the coating matrix, giving it a reticulated pore structure. The X-ray diffraction analysis revealed the formation of a new prevalent phase—forsterite (Mg_2_SiO_4_). Peaks corresponding to magnesium and periclase (MgO), as well as rutile (TiO_2_), were also observed in the diffraction patterns of the resulting coatings. The coatings demonstrated very high adhesion strength (critical load equaled to 24–35 N) compared to diatomite-based coatings without the addition of TiO_2_ particles (critical load <10 N). Diatomite-based coatings, the composition of which included TiO_2_ particles, had 2–3 orders of magnitude greater polarization resistance than coatings without the addition of titanium dioxide particles. The in vitro immersion method was implemented to study the dissolution rate of both the coated and uncoated samples, which showed that the dissolution rate of all coated samples, regardless of the deposition voltage, has a linear character. The mass loss rate of the uncoated Mg alloy samples exceeds that of the coated samples after 4 days of immersion and amounts to more than 6% at the end of the experiment. NIH/3T3 mouse fibroblasts were used to assess the in vitro cytotoxicity of the coatings, i.e., cell viability, during direct contact with the coating. The number of viable cells after direct contact with the coatings was 94–98%, which is not a statistically significant difference from the control samples. Meanwhile, the number of viable cells in contact with pure magnesium alloy was only 43%. Thus, modification of the surface of magnesium alloy samples by means of micro-arc coatings based on diatomite with TiO_2_ particles yielded an increase in their cytocompatibility by more than two-fold.

A comparative analysis of the properties of the coatings deposited at different stresses of the MAO process was carried out, and it was concluded that the coatings formed at 350 and 450 V are the most promising for practical application in terms of the set of optimal properties. These coatings are characterized not only by sufficient thickness and roughness and high adhesive strength, but also by the highest corrosion resistance, exceeding that in the works of other authors [56,57]. They had the highest values of polarization resistance at 1.22 × 10^8^ Ω cm^2^ and 1.41 × 10^8^ Ω cm^2^, respectively, and the lowest values of corrosion current density at 3.3 × 10^−10^ A cm^–2^ and 4.0 × 10^−10^ A cm^−2^, respectively.

## Figures and Tables

**Table 1:** Chemical composition of the coatings, at. %.

Element	350 V	400 V	450 V	500 V
O Kα	58.7 ± 2.5	62.2 + 1.7	60.7 ± 1.7	61.3 ± 0.6
F Kα	4.1 ± 0.9	3.9 ± 1.3	5.2 ± 0.8	4.72 ± 0.5
Na Kα	1.4 ± 0.4	1.8 ± 0.2	2.1 ± 0.6	2.1 ± 0.3
Mg Kα	14.8 ± 1.8	12.9 ± 1.3	14.3 ± 2.6	14.49 ± 0.6
Al Kα	0.9 ± 0.1	0.8 ± 0.0	0.8 ± 0.1	0.84 ± 0.0
Si Kα	17.2 ± 1.2	15.6 ± 0.4	14.7 ± 0.6	14.8 ± 0.2
Ti Kα	2.9 ± 0.2	2.6 ± 0.7	2.2 ± 0.6	1.82 ± 0.2

**Table 2:** Chemical composition of the coatings (cross-section), at. %.

Element	350 V	400 V	450 V	500 V
O Kα	57.2 ± 1.5	59.8 ± 0.4	62.4 ± 1.3	61.6 ± 2.5
F Kα	8.0 ± 0.8	5.6 ± 1.0	4.1 ± 0.9	3.8 ± 0.7
Na Kα	0.5 ± 0.08	1.0 ± 0.1	2.9 ± 0.8	3.0 ± 0.7
Mg Kα	14.5 ± 1.6	14.6 ± 1.0	9.7 ± 0.8	11.0 ± 3.6
Al Kα	0.4 ± 0.2	0.7 ± 0.1	0.6 ± 0.1	0.6 ± 0.0
Si Kα	17.5 ± 0.1	16.7 ± 0.5	18.5 ± 1.2	17.8 ± 2.1
Ti Kα	1.7 ± 0.3	1.4 ± 0.6	1.5 ± 0.3	2.0 ± 1.0

**Table 3:** Electrochemical parameters of the Mg alloy and coated samples.

Sample	Diatomite + TiO_2_		
	E_c_, V	I_c_, A cm^−2^	R_p_, Ω cm^2^
Mg alloy	−1.43	2.25 × 10^−6^	1.61 × 10^4^
350 V	−0.46	3.30 × 10^−10^	1.22 × 10^8^
400 V	−0.02	4.01 × 10^−8^	1.65 × 10^6^
450 V	−0.52	4.01 × 10^−10^	1.41 × 10^8^
500 V	−0.36	1.32 × 10^−8^	4.21 × 10^6^

## Data Availability

Not applicable.
